# Examining Cardiomyocyte Dysfunction Using Acute Chemical Induction of an Ageing Phenotype

**DOI:** 10.3390/ijms21010197

**Published:** 2019-12-27

**Authors:** Said Masoud, Fraser McDonald, Dirk Bister, Claire Kotecki, Martin D. Bootman, Katja Rietdorf

**Affiliations:** 1 School of Life, Health and Chemical Sciences, The Open University, Walton Hall, Milton Keynes MK7 6AA, UKclaire.kotecki@open.ac.uk (C.K.); katja.rietdorf@open.ac.uk (K.R.); 2 Department of Orthodontics, Kings College London, 310 Tower Wing, Guy’s, London SE1 9RT, UK; fraser.mcdonald@kcl.ac.uk (F.M.); d.bister@doctors.org.uk (D.B.)

**Keywords:** cardiac, ageing, calcium, remodelling, alternans, arrhythmia

## Abstract

Much effort is focussed on understanding the structural and functional changes in the heart that underlie age-dependent deterioration of cardiac performance. Longitudinal studies, using aged animals, have pinpointed changes occurring to the contractile myocytes within the heart. However, whilst longitudinal studies are important, other experimental approaches are being advanced that can recapitulate the phenotypic changes seen during ageing. This study investigated the induction of an ageing cardiomyocyte phenotypic change by incubation of cells with hydroxyurea for several days ex vivo. Hydroxyurea incubation has been demonstrated to phenocopy age- and senescence-induced changes in neurons, but its utility for ageing studies with cardiac cells has not been examined. Incubation of neonatal rat ventricular myocytes with hydroxyurea for up to 7 days replicated specific aspects of cardiac ageing including reduced systolic calcium responses, increased alternans and a lesser ability of the cells to follow electrical pacing. Additional functional and structural changes were observed within the myocytes that pointed to ageing-like remodelling, including lipofuscin granule accumulation, reduced mitochondrial membrane potential, increased production of reactive oxygen species, and altered ultrastructure, such as mitochondria with disrupted cristae and disorganised myofibres. These data highlight the utility of alternative approaches for exploring cellular ageing whilst avoiding the costs and co-morbid factors that can affect longitudinal studies.

## 1. Introduction

Ageing studies are typically conducted via protracted longitudinal experiments that compare cells, tissues or samples from young and old animals, or by using progeroid models. Although these approaches have provided many significant insights into the processes and outcomes of ageing, they can be expensive and time-consuming as they require prolonged maintenance of cell/animal stocks. Moreover, it may be difficult to ensure that the environment, nutrition and condition of animals are exactly alike over protracted periods, and that co-morbid factors do not impinge the presumed ageing phenotypes observed [1]. For these reasons, it would be particularly useful to establish acute models of ageing that do not require aged cells or animals.

A key purpose of ageing studies is to contrast the biology of young versus old cells and tissues. From such studies, it is possible to understand the morphological and functional alterations that underpin age-dependent cell and organ dysfunction, which consequently lead to frailty and morbidity [2]. Whilst aged cells, tissues and animals are commonly used to study ageing, they are not the only experimental paradigms that can be applied. Indeed, any approach that can recapitulate the remodelling of cells that occurs during ageing has validity. A plausible alternative to protracted longitudinal experiments is to acutely induce changes in cellular form and function that replicate those seen in aged tissues. This can be achieved, for example, by culturing cells ex vivo for prolonged periods, using iPSC-derived cells from patient samples, or via chemically-induced cellular remodelling [1,3,4,5,6].

Previous studies have shown that acute treatment with hydroxyurea (HU) can induce cellular senescence and ageing phenotypes in both isolated cells [7,8,9] and whole animals [10]. For example, HU was used to establish a model of neuronal ageing, using neural stem cells [6]. HU is widely used in the treatment of sickle cell anaemia and as a tumour therapeutic agent, and has been shown to have a number of cellular effects including inhibition of DNA synthesis, growth arrest by inducing cell cycle inhibitors and senescence. The pleiotropic effects of HU lead to a cellular phenotype that has many similarities to that observed in physiological ageing in animals or cells [7,11], although further studies are required to verify the utility of this model in different cell and tissue types.

The heart is a muscular organ that beats over a billion times in a typical human lifespan. Each heartbeat involves the synchronized contraction of atrial and ventricular chambers in a process known as the ‘cardiac cycle’ [12]. Within each cardiomyocyte, cytosolic calcium (Ca^2+^) signals act as the mediators between the electrical depolarisation of the cell and excitation–contraction coupling (EC-coupling) that results in contraction of the heart and blood flow. Maintenance of the heart’s structure and Ca^2+^ homeostasis are crucial for its function: a precise sequence of electrical, ionic and mechanical events is necessary for each cardiomyocyte to contract and recover [13]. During ageing, or in disease states such as atrial fibrillation (AF), remodelling of cellular components and Ca^2+^ signalling can occur, resulting in deficient cardiac output [14,15,16,17,18,19,20]. Mistiming of cytosolic Ca^2+^ signals, for example, alters the rhythmic contraction of cardiomyocytes by making cells refractory to incoming electrical signals [21]. Even modest changes in Ca^2+^ signalling, if persistent, can gradually alter cardiomyocyte function [22]. Moreover, other ageing-related changes, such as increased oxidative stress, can be involved in the pathogenesis of AF [23]. It is widely accepted that ageing is a major risk factor for the development of AF due to structural and functional remodelling [24,25,26,27,28], but the precise changes in cardiomyocyte function that underlie such deleterious outcomes are not fully known.

The experiments described in this study sought to explore whether an acute, chemically-induced, cellular model of ageing could be established for cardiomyocytes. For the reasons given above, recapitulating the ageing-dependent changes in the form and function of cardiomyocytes in an acute model would be beneficial in many ways. In this study, neonatal ventricular myocytes (NRVMs) were treated continuously with HU for periods of up to 7 days. After which the cells were examined for both markers of ageing and functional/morphological status. Specifically, we compared Ca^2+^ signalling, mitochondrial function, ROS production, autophagy and structural changes between control and HU-treated NRVMs. Our data indicate that is it possible to recapitulate aspects of ageing through acute chemical treatment of cardiomyocytes, and this approach may provide a novel reproducible and tractable alternative to more expensive experiments involving animal cohorts.

## 2. Results

### 2.1. Altered Ca^2+^ Signalling in HU-Treated NRVMs

**Figure 1:**
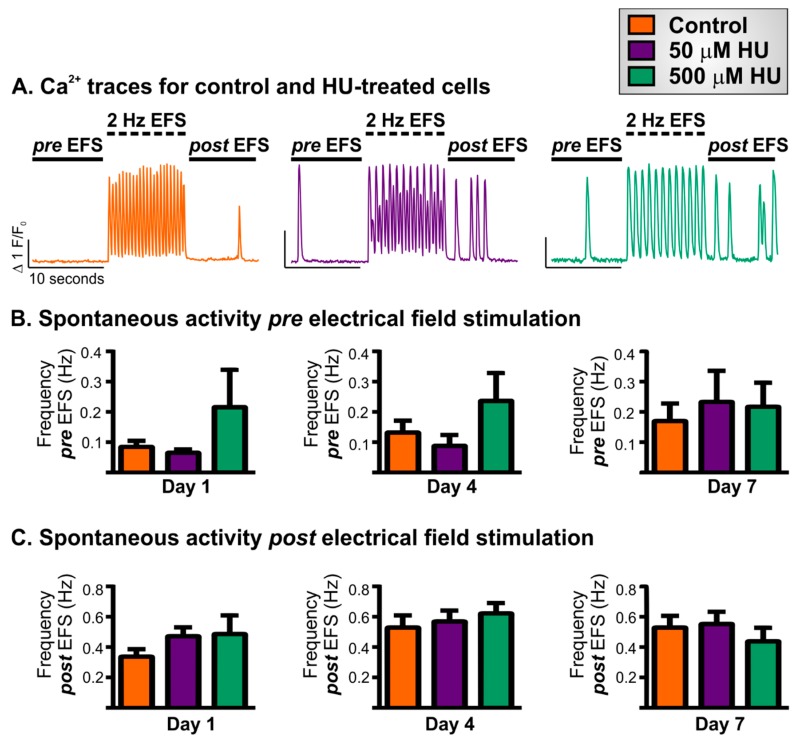
Effect of hydroxyurea (HU) treatment on spontaneous Ca^2^⁺ signals in neonatal ventricular myocytes (NRVMs). Panel (**A**) illustrates the imaging and EFS stimulation protocol used and shows representative Ca^2^⁺ traces obtained from control NRVMs, and cells incubated with 50 or 500 µM HU for 4 days. Panel (**B**) quantifies the average frequency of spontaneous Ca^2+^ signals *pre* EFS for control NRVMs and for cells incubated with 50 or 500 µM HU for 1, 4 or 7 days. Panel (**C**) quantifies the average frequency of spontaneous Ca^2+^ signals *post* EFS for control NRVMs and for cells incubated with 50 or 500 µM HU for 1, 4 or 7 days. The data are presented as mean ± S.E.M. and were analysed using one-way ANOVA. The data were obtained from nine replicate NRVM coverslips for each condition.

The effects of HU incubation on Ca^2^⁺ homeostasis and cytosolic Ca^2^⁺ signalling were examined by measuring the frequency of spontaneous Ca^2+^ signals in resting NRVMs, and the ability of NRVMs to follow electrical pacing (2 Hz electrical field stimulation; EFS; see Materials and Methods). Cardiomyocytes display spontaneous cytosolic Ca^2^⁺ signals due to stochastic openings of ryanodine receptors (RyRs). Changes in the frequency of spontaneous Ca^2^⁺ signals can reflect modified RyR expression and open probability (e.g., via covalent modifications) or alterations in the Ca^2^⁺ concentration with the lumen of the sarcoplasmic reticulum (SR; the major Ca^2^⁺ store in muscle cells) [29]. To visualise cytosolic Ca^2+^ signals, control and HU-treated NRVMs were loaded with Cal-520 and imaged at 12 Hz using a widefield microscope, which was sufficient to resolve both spontaneous Ca^2+^ signals and those triggered by the application of EFS. The experimental protocol used for these experiments is illustrated by the traces in Figure 1A. The imaging experiments lasted 30 s and were performed as follows:

1. Ten-second recording of spontaneous activity (‘*pre* EFS’).
2. Ten-second recording during 2 Hz EFS (‘EFS’).
3. Ten-second recording of spontaneous activity (‘*post* EFS’).

This protocol was applied on days 1, 4 and 7 following the addition of either 50 or 500 µM HU to the NRVM growth medium. NRVMs not treated with HU, but cultured for the same length of time, were used as controls. The first part of the recording (*pre* EFS) established the spontaneous Ca^2+^ signal activity in the cells prior to the application of EFS pacing. The second phase of the recording (EFS), examined whether the HU treatment of the cells affected their ability to follow electrical pacing. Whilst the third part of the recording (*post* EFS) assessed whether the electrical pacing affected the spontaneous Ca^2+^ signal activity in the cells.

The Ca^2+^ traces shown in Figure 1A were derived from representative NRVMs on day 4 of HU incubation. In all three conditions (control, 50 and 500 µM HU), NRVMs displayed a low frequency of spontaneous Ca^2+^ signals (typically <1 Hz). The majority of spontaneous Ca^2+^ signals were observed to be Ca^2+^ waves, which initiated in a subcellular region of an NRVM and then propagated either wholly or partially throughout the rest of the cell. There were no significant effects of HU treatment on spontaneous Ca^2+^ signals at either of the concentrations used (Figure 1B). For all cells, the frequencies of spontaneous Ca^2+^ signals increased after the application of EFS (Figure 1C), most likely due to an acutely increased SR Ca^2+^ loading during the burst of electrical stimulation. However, there was no statistically significant effect of HU treatment on the frequency of spontaneous Ca^2+^ signals in the *post* EFS recording periods.

The application of EFS causes rapid Ca^2+^ signals within the NRVMS via the triggering of the EC-coupling machinery. In the course of these experiments, the cellular responses to EFS were classified into two types:

1. Regular responses. Ca^2+^ transients that were elicited by every EFS pulse, and had similar pulse-to-pulse amplitudes.
2. Alternans. Cells responded to every EFS pulse, but the amplitude of the Ca^2+^ transients alternated between large and small.

**Figure 2:**
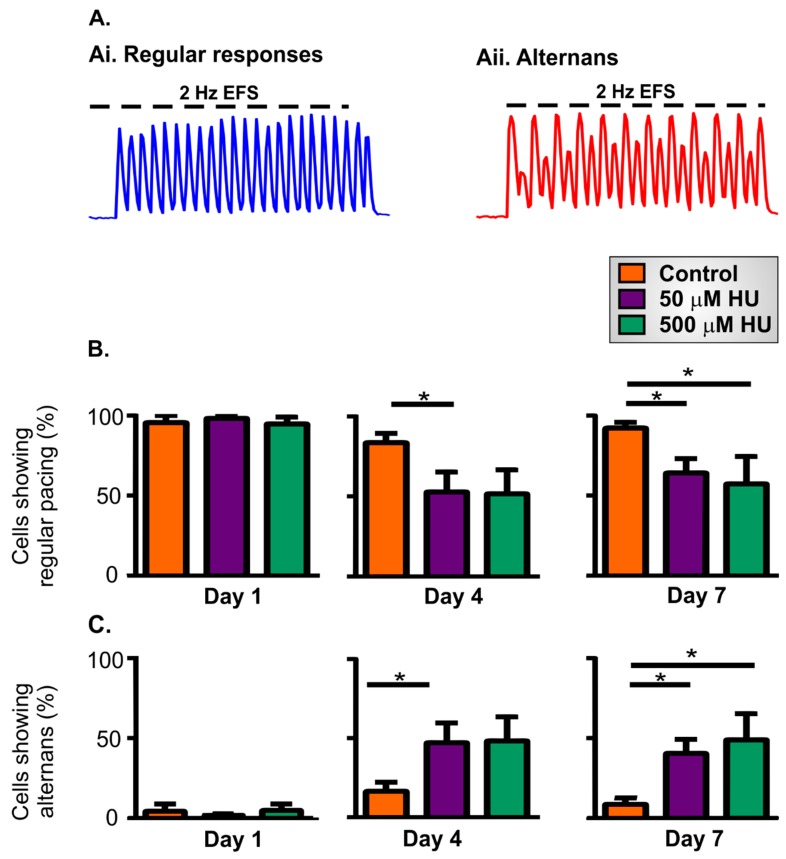
Responses to electric field stimulation (EFS) in control or HU-treated NRVMs. Panel **A** illustrates regular responses during EFS application (**Ai**) and alternans (**Aii**). Panel (**B**) shows the percentage of NRVMs that displayed regular responses to EFS on different days of incubation with HU. Panel (**C**) illustrates the percentage of NRVMs that responded to EFS with alternans. The data are presented as mean ± S.E.M. and were analysed using one-way ANOVA. * denotes *p* < 0.05. The data were obtained from nine replicate NRVM coverslips for each condition.

Examples of these different types of responses are shown in Figure 2A.

**Figure 3:**
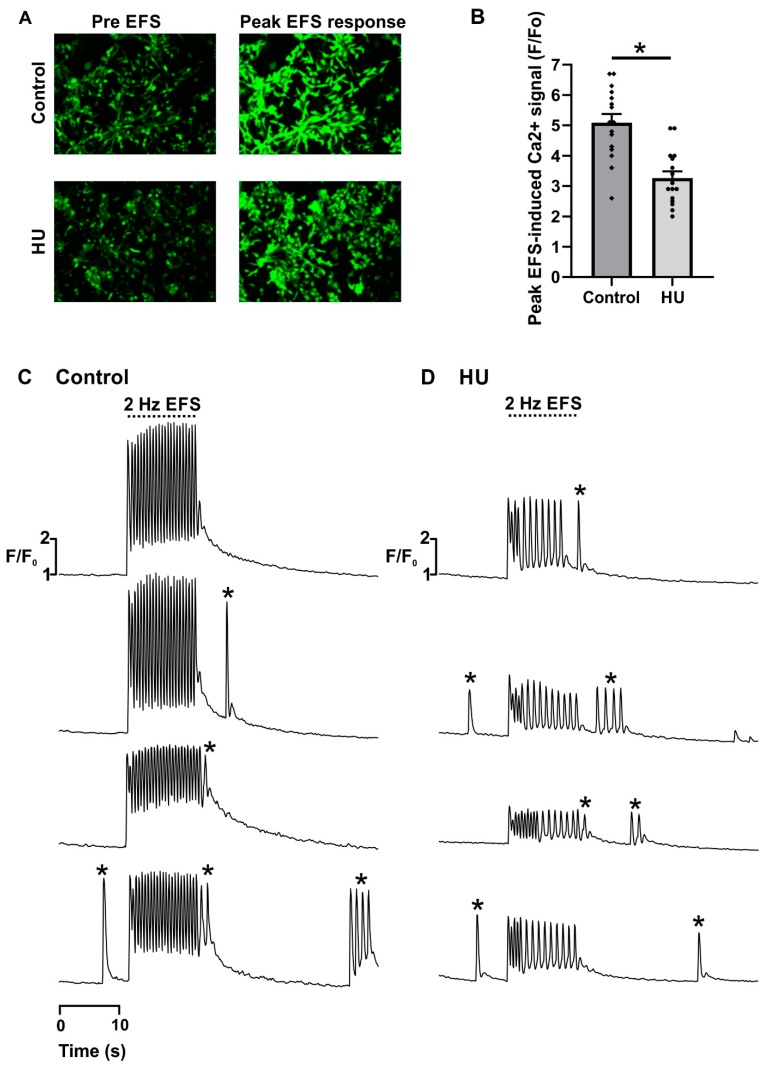
Responses to electric field stimulation (EFS) in control or HU-treated NRVMs. Panel (**A**) shows images of control and HU-treated NRVMs loaded with Cal-520 (day 7 of HU incubation). Images of diastolic (*pre* EFS) and systolic (*peak* EFS response) Cal-520 fluorescence are shown. Panel (**B**) shows quantitation of the peak systolic response to EFS in control and HU-treated cells. Panels (**C**) and (**D**) show illustrative responses of control and HU-treated cells to EFS (the EFS was applied during the time shown by the dashed line). The responses shown in Panels (**C**,**D**) were obtained by averaging the Cal-520 signal across a whole field of cells (i.e., fields of cells similar to those in Panel (**A**)). Averaging across a whole field of cells does not allow visualisation of the spontaneous Ca^2+^ transients that occur within individual cells (due to the asynchronous nature of spontaneous Ca^2+^ transients between cells). However, spontaneous action potentials that generate simultaneous Ca^2+^ signals in all the cells in a field of view can be resolved. Examples of spontaneous action potential-evoked Ca^2+^ transients (marked with *) have the same amplitude as those triggered by EFS. The data were obtained from nine replicate NRVM coverslips for each condition.

Almost all NRVMS, whether control or HU-treated, showed regular pacing when applying 2 Hz EFS on day 1 (Figure 2B). Most control cells also showed regular responses to EFS application on days 4 and 7 (Figure 2B). In contrast, the number of HU-treated NRVMs that showed regular responses to EFS pulses was reduced on days 4 and 7 of HU incubation (Figure 2B). On day 1, neither control nor HU-treated cells showed a high incidence of alternans (Figure 2C). In contrast, the occurrence of alternans significantly increased in NRVMs treated with HU on days 4 and 7 (Figure 2C). The observation that control and HU-treated NRVMs responded similarly to EFS application on day 1 indicates that the effects HU were not simply due to the presence of the compound (for 24 h), but rather that they appeared because of cellular remodelling over several days. In addition to the decreased fidelity of EFS-induced pacing shown in Figure 2, the HU-treated NRVMs displayed reduced systolic Ca^2+^ responses (Figure 3). Cal-520 loaded similarly into the control and HU-treated cells (Figure 3A), but the peak systolic Ca^2+^ transients observed during the application of EFS were significantly lower following HU treatment (Figure 3B,C).

The decreased ability of HU-treated NRVMs to follow EFS pacing, as well as the reduced systolic Ca^2+^ signals, pointed to a change in the processes involved in EC-coupling. A key factor leading to dysregulated Ca^2+^ homeostasis in cardiac myocytes is altered sarco-endoplasmic reticulum Ca^2+^ ATPase (SERCA) activity and a consequent decline in the SR Ca^2+^ content, both of which are known to occur during heart failure. To test whether this occurred in HU-treated NRVMs, the SR Ca^2+^ content was measured in control and HU-treated cells. For these experiments, the SR Ca^2+^ store was emptied using caffeine, and the characteristics of the caffeine-induced Ca^2+^ signal were assessed. Caffeine activates RyRs such that they can rapidly deplete the entire SR of Ca^2+^, thus providing a means to assay the total SR Ca^2+^ content. The imaging experiment lasted 80 s and was performed by continuously recording the following three parts as follows:

1. Twenty-second recording of spontaneous Ca^2+^ signals in resting cells to ensure that cells were healthy and showing typical levels of spontaneous activity.
2. Thirty-second superfusion with Ca^2+^-free imaging buffer supplemented with the Ca^2+^ chelator EGTA (500 µM).
3. Thirty-second superfusion with 1 mM caffeine in Ca^2+^-free imaging buffer supplemented with EGTA.

**Figure 4:**
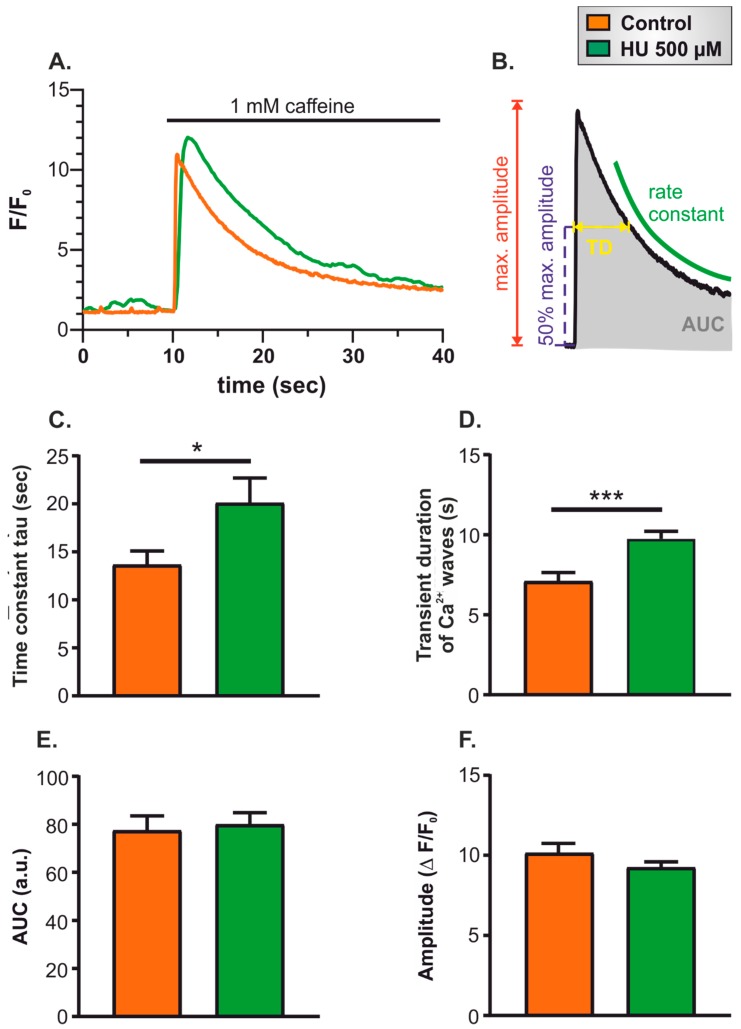
Comparison of caffeine-induced Ca^2+^ transients in control and HU-treated NRVMs. Panel (**A**) shows representative caffeine-induced Ca^2+^ transients in control and HU-treated cells. Panel (**B**) illustrates the parameters quantified to characterise the caffeine-induced Ca^2+^ signals. Panel (**C**) shows the quantification of the time constant, tau, derived from fitting mono-exponential decay curves to the caffeine-induced Ca^2+^ signals. Panel (**D**) shows the quantification of the transient duration measured as full-width at half-maximal amplitude. Panel (**E**) shows the quantification of the integrated Ca^2+^ signal (area under the curve; ‘AUC’). Panel (**F**) indicates the maximum amplitude (ΔF/F_0_) of caffeine-induced Ca^2+^ transients in control and 500 µM HU-treated cells. The data shown were all obtained on day 4 of HU incubation. The data are presented as mean ± S.E.M. and were analysed using unpaired *t*-tests. * and *** denote *p* < 0.05 and *p* < 0.001, respectively. The data were obtained from seven replicate NRVM coverslips for each condition (see Materials and Methods for details).

The application of caffeine caused a rapid, transient increase of cytosolic Ca^2+^ concentration due to the activation of RyRs on the SR (Figure 4A). The parameters that were quantitated to characterise the caffeine-induced Ca^2+^ signal are illustrated in Figure 4B. The rate of Ca^2+^ clearance was characterised by fitting a mono-exponential decay curve, and by measuring the width of the Ca^2+^ signal at half-maximal amplitude (Figure 4B; TD, ‘transient duration’). In addition, the integrated caffeine-induced Ca^2+^ signal (hereafter denoted ‘area under the curve’; AUC) was used as an estimate for the total SR Ca^2+^ content [30,31].

The time constant for recovery of the cytosolic Ca^2+^ signal and the Ca^2+^ signal duration (measured at half-maximal amplitude) (Figure 4C,D) were both significantly increased in HU-treated NRVMs. These data indicate that the HU treatment slowed the rate of decay of the Ca^2+^ signal. In contrast, neither the AUC nor the maximum amplitude of the Ca^2+^ signal was different between control and HU-treated NRVMs (Figure 4E,F). It, therefore, appears that HU treatment did not affect steady-state SR Ca^2+^ content, but diminished the ability of cells to reverse cytosolic Ca^2+^ signals.

### 2.2. Altered Mitochondrial Metabolism in HU-Treated NRVMs

**Figure 5:**
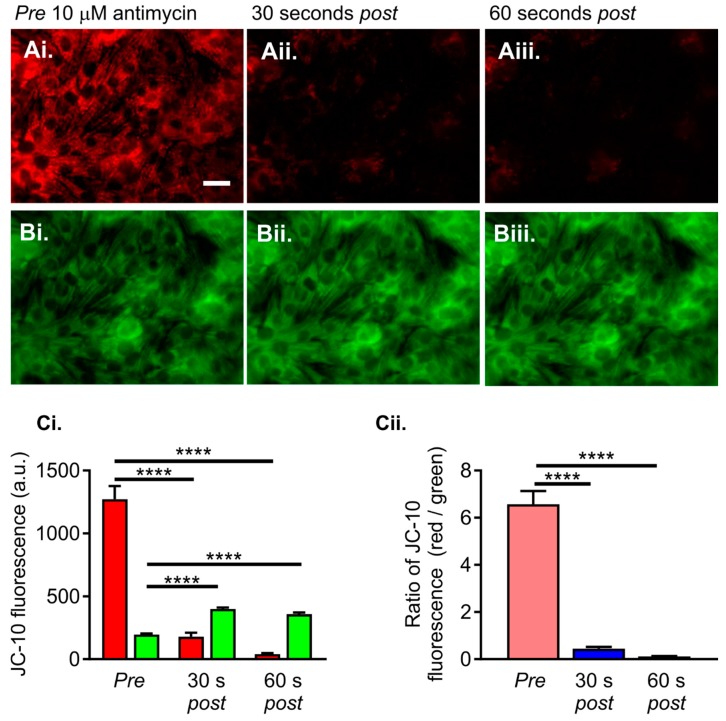
Using the ratiometric fluorescent indicator JC-10 to determine mitochondrial membrane potential in NRVMs. Panel (**A**) shows the red fluorescence of JC-10 aggregates before (**Ai**) and after (**Aii**,**Aiii**) addition of 10 µM antimycin. Panel (**B**) shows the green fluorescence of JC-10 monomers before (**Bi**) and after (**Bii**,**Biii**) addition of antimycin. Panel (**C**) illustrates the fluorescence (a.u.) of JC-10 monomers (green bars) and aggregates (red bars) before *(pre)* and after *(post)* addition of antimycin. The ratio of the red/green JC-10 fluorescence is presented in Panel Cii. The data are presented as mean ± S.E.M. and were analysed using one-way ANOVA. **** denotes *p* < 0.0001. The data were obtained from 16 replicate NRVM coverslips for each condition (see Materials and Methods for details). The scale bar represents 20 µm.

The mitochondrial membrane potential in the NRVMs was quantified using the ratiometric fluorescent indicator JC-10 [32,33]. This indicator emits green or red fluorescence when excited with 488 nm light. The colour of the emitted light depends on the presence of monomeric or aggregated forms of JC-10 within the mitochondrial matrix. In normally respiring cells, JC-10 accumulates within mitochondria and forms aggregates that have a red fluorescence emission. An example of the accumulation of red-emitting JC-10 aggregates within NRVMs is shown in Figure 5Ai. The JC-10 aggregates are in equilibrium with JC-10 monomers, which have a green fluorescence emission. An example of the green emission from JC-10 monomers within the same cells is shown in Figure 5Bi.

Depolarisation of the mitochondrial membrane potential, for example, using antimycin, causes the red-emitting JC-10 aggregates to dissipate into green-emitting JC-10 monomers. An example of the effects of antimycin (10 µM) on the red and green emission profiles of JC-10 is depicted in Figure 5A,B. It is evident that the addition of antimycin caused a rapid decrease in the red emission, whilst simultaneously increasing the intensity of the green emission. JC-10 can, therefore, be used to monitor mitochondrial membrane potential by assessing either the red or green fluorescence emission (Figure 5Ci) or by calculating the ratio of red to green fluorescence (Figure 5Cii).

**Figure 6:**
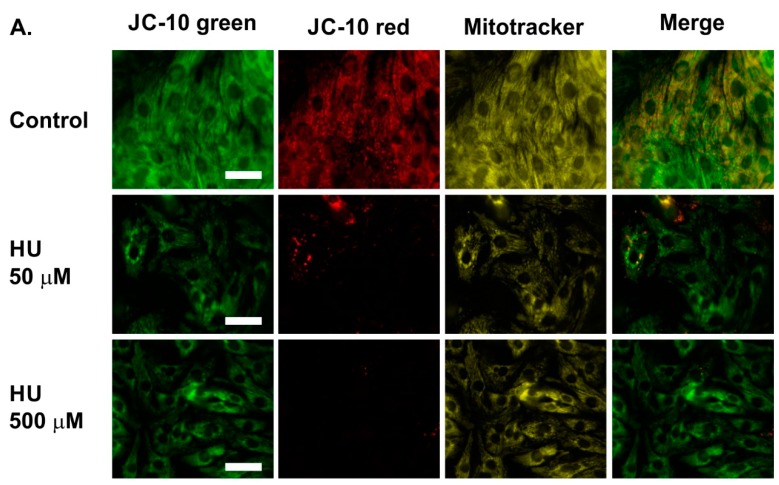

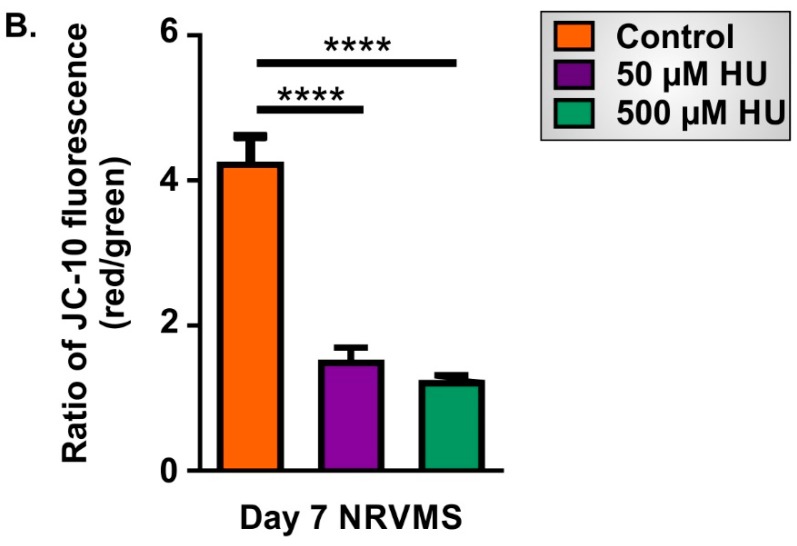
Assessment of the mitochondrial membrane potential in control and HU-treated NRVMs using JC-10. Panel (**A**) shows representative JC-10 and MitoTracker Red fluorescence images obtained using NRVMs on day 7. MitoTracker Red was used to confirm that the JC-10 staining was localised in mitochondria and to choose regions of interest for analysis. The scale bar represents 10 µm. Panel (**B**) shows a quantitative summary of the JC-10 red:green ratio in Day 7 control and HU-treated NRVMs. The data are presented as mean ± S.E.M. and were analysed using one-way ANOVA. **** denotes *p* < 0.0001. The data were obtained from nine replicate NRVM coverslips for each condition.

JC-10 was used to compare the mitochondrial membrane potential in control and HU-treated NRVMs (Figure 6). For these experiments, cells were co-loaded with MitoTracker Red (>650 nm emission to avoid overlap with JC-10). The MitoTracker fluorescence was used to focus on the NRVMs so that imaging regions could be chosen in an unbiased manner and to avoid photobleaching of the JC-10 fluorescence prior to the image acquisition. After focussing on the cells, images for the green and the red JC-10 fluorescence emission, and the MitoTracker fluorescence emission, were obtained. The representative images in Figure 6A show NRVMs on day 7. Control cells displayed substantial green and red JC-10 fluorescence emission profiles (Figure 6A, top row). In contrast, NRVMs incubated with HU showed almost no red JC-10 fluorescence emission and a lesser green fluorescence (Figure 6A, middle and bottom rows). To quantitate the JC-10 fluorescence, five regions per coverslip were chosen by their MitoTracker Red staining, and the ratio of JC-10 green and red emission in those regions was determined. Quantification of the JC-10 fluorescence indicated that incubation with either 50 µM or 500 µM HU caused a significant reduction in the JC-10 red:green fluorescence emission ratio on day 7 of the HU treatment (Figure 6B).

These data support the hypothesis that there was a reduction in mitochondrial membrane potential in the HU-treated NRVMs. In addition to the reduced JC-10 red:green emission ratio, there was a reduction in the absolute uptake of both JC-10 and MitoTracker Red in the HU-treated NRVMs (Figure 6A). Both JC-10 and MitoTracker Red load into mitochondrial in a membrane potential-dependent manner. A lesser uptake of these compounds concurs with the notion that HU reduced the mitochondrial membrane potential within the HU-treated cells.

### 2.3. Altered Reactive Oxygen Species (ROS) Production in HU-Treated NRVMs

The results presented above indicate that HU incubation led to reduced mitochondrial membrane potential. Depolarised mitochondria have been shown to have an increased ROS production [34]. Therefore, the effect of HU on ROS production in the NRVMs was assessed using the fluorescent indicator ROS Brite. The experimental protocol for these experiments was as follows:

1. Ten-second recording of ROS Brite fluorescence in control or HU-treated NRVMs.
2. Addition of 10 µM antimycin, followed by further imaging of ROS Brite fluorescence for 120 s.

**Figure 7:**
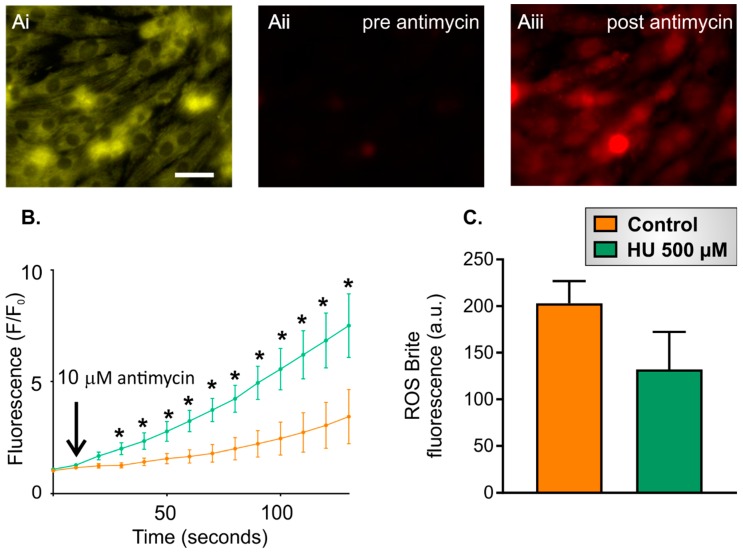
ROS production in control and HU-treated NRVMs. Panel (**Ai**) shows an image of NRVMs stained with MitoTracker Red, which was used to focus on the cells at the start of the experiment and to find unbiased regions of interest to analyse ROS Brite fluorescence. Panels (**Aii**) and (**Aiii**) show ROS Brite fluorescence in the same cells *pre* and 120 s *post* antimycin treatment, respectively. The scale bar represents 20 µm. Panel (**B**) shows changes in the ROS Brite fluorescence *pre* and *post* antimycin addition in control and 500 µM HU-treated cells on day 4 post-HU treatment. The data are presented as mean ± S.E.M. and were analysed using unpaired *t*-tests for the individual time points. Panel (**C**) compares the basal ROS levels in control and 500 µM HU-treated cells. The data are presented as mean ± S.E.M. and were analysed using an unpaired *t*-test. * denotes *p* < 0.05. The data were obtained from five replicate NRVM coverslips for each condition.

NRVMs incubated for 7 days with 500 µM HU were used for this experiment, as this treatment significantly altered the mitochondrial membrane with respect to control cells (Figure 6). NRVMs were co-loaded with MitoTracker Red to identify the cells and choose mitochondrial regions of interest to analyse (Figure 7Ai) since ROS Brite only showed a minimal fluorescence at the initiation of experiments (Figure 7Aii). To quantitate ROS production in the NRVMs, two parameters were measured:

1. Basal ROS level. This was calculated as the mean ROS Brite fluorescence in the first five images captured during an imaging experiment.
2. Inducible ROS production. This was calculated following the addition of antimycin to the cells by measuring the mean ROS Brite fluorescence in 10-s intervals over the 120-s experimental period. An example of ROS Brite cellular fluorescence in cells treated with antimycin is shown in Figure 7Aiii.

As shown in Figure 7B, the ROS Brite fluorescence progressively increased after the addition of antimycin and was significantly greater in HU-treated NRVMs compared to the control cells over the course of the recording. The basal ROS levels at the start of the experiment were not significantly different between control and HU-treated cells (Figure 7C).

### 2.4. Increased Autophagy in HU-Treated NRVMs

**Figure 8:**
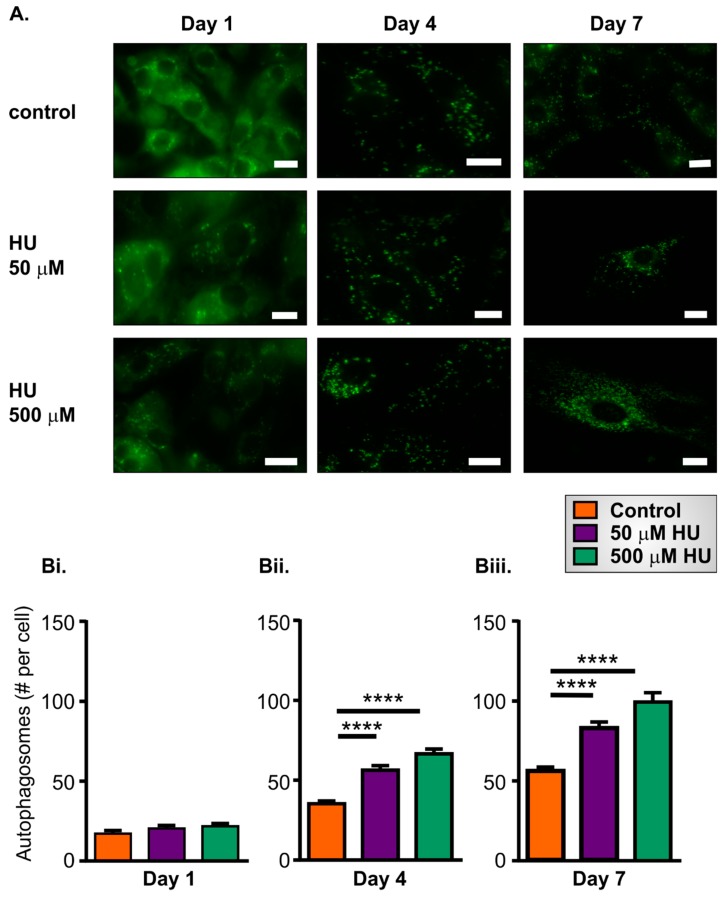
Autophagy in control and HU-treated NRVMs. Panel (**A**) shows representative images of autophagosomes stained with a CytoID kit in control and HU-treated cells. The scale bar represents 10 µm. Panels (**Bi**–**Biii**) show quantitation of autophagosome numbers in control cells and HU-treated NRVMs on days 1, 4 and 7. The data are presented as mean ± S.E.M. and were analysed using one-way ANOVA. **** denotes *p* < 0.0001, respectively. The data were obtained from nine replicate NRVM coverslips for each condition.

Autophagy is a mechanism by which cells recycle damaged and dysfunctional organelles and other molecules, or respond to bioenergetic demands by breaking down cellular constituents to provide resources necessary for anabolic processes. A routine method for quantifying autophagy is to count the number of autophagic vesicles within a cell after labelling them with a fluorescent probe [35,36]. To assess autophagy in the NRVMs, cells were loaded with CytoID reagent for 30 min at 37 °C degrees, and images were subsequently acquired using widefield fluorescence microscopy. After staining with the CytoID reagent, the autophagosomes within NRVMs were evident as green fluorescent punctae (Figure 8A). The number of autophagosomes was similar in control and HU-treated cells on day 1 (Figure 8Bi) but increased significantly on days 4 and 7. The increase in the number of autophagosomes was more pronounced in the HU-treated cells (Figure 8Bii,Biii).

### 2.5. Ultrastructural Changes in HU-Treated NRVMs

**Figure 9:**
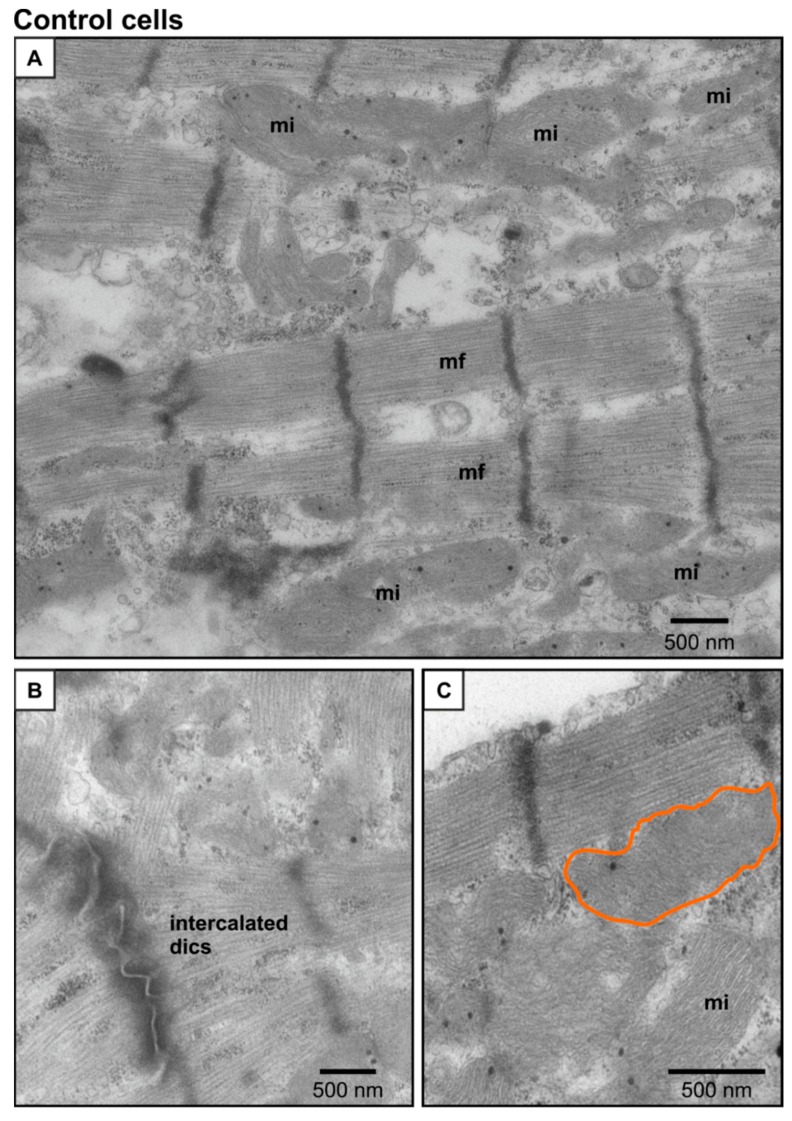

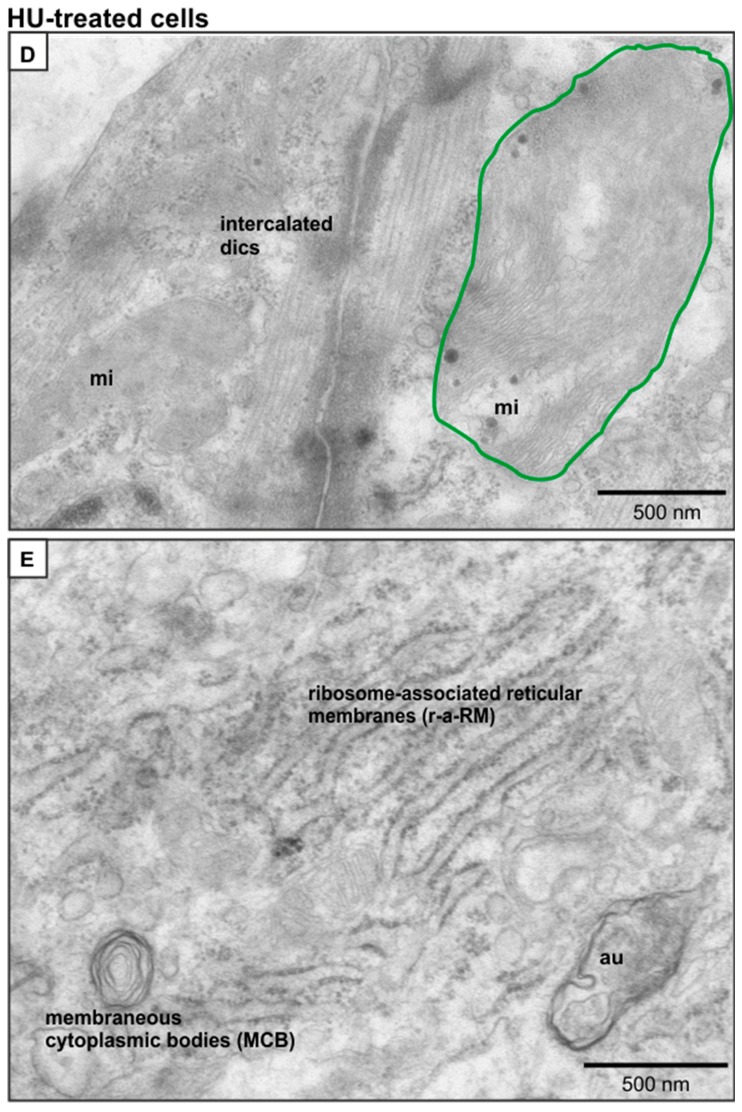
Transmission electron microscopy (TEM) images of control and HU-treated cells. Panels (**A**–**C**) show representative TEM images of control cells on day 7, illustrating the presence of myofilaments (mf), intercalated discs and mitochondria (mi) with preserved cristae (outlined in orange). Panels (**D**,**E**) are representative images from 500 µM HU-treated cells on day 7, showing the presence of mitochondria with disrupted cristae, outlined in green (Panel (**D**)), ribosome-associated reticular membranes (r-a-RM), membranous cytoplasmic bodies (MCB) and autophagosomes (au). The data were selected from 35 images for each condition.

Transmission electron microscopy (TEM) images of control day 7 NRVMs (Figure 9A–C) identified the typical structures seen in cardiac myocytes: myofilaments with clear z-lines (Figure 9A), abundant mitochondria in between the myofilaments (Figure 9A) and intercalated disks between the myocytes (Figure 9B). The mitochondria showed clearly defined cristae (Figure 9C). NRVMs treated with 500 µM HU for 7 days exhibited the same characteristic cardiac myocyte structures with z-lines, myofilaments and intercalated discs (Figure 9D). However, they also contained structures that were rarely seen in the control cells (Figure 9E). In particular, there was an increase in the abundance of reticular membranes decorated with ribosomes (Figure 9D). These ribosome-associated reticular structures appeared around the sarcolemma and perinuclear area of the HU-treated NRVMs. Moreover, the HU-treated cells possessed evident double-membrane autophagosomes, and membranous cytoplasmic bodies known as lipofuscin granules, which are circular lipid aggregates linked to ganglioside accumulation. Lipofuscin granules have been shown to accumulate in aged tissues or because of lysosomal storage diseases ([37]; Figure 9E). The increased appearance of autophagosomes in TEM images from HU-treated cells accords with the data obtained using the fluorescent CytoID stain in living NRVMs (Figure 8).

**Figure 10:**
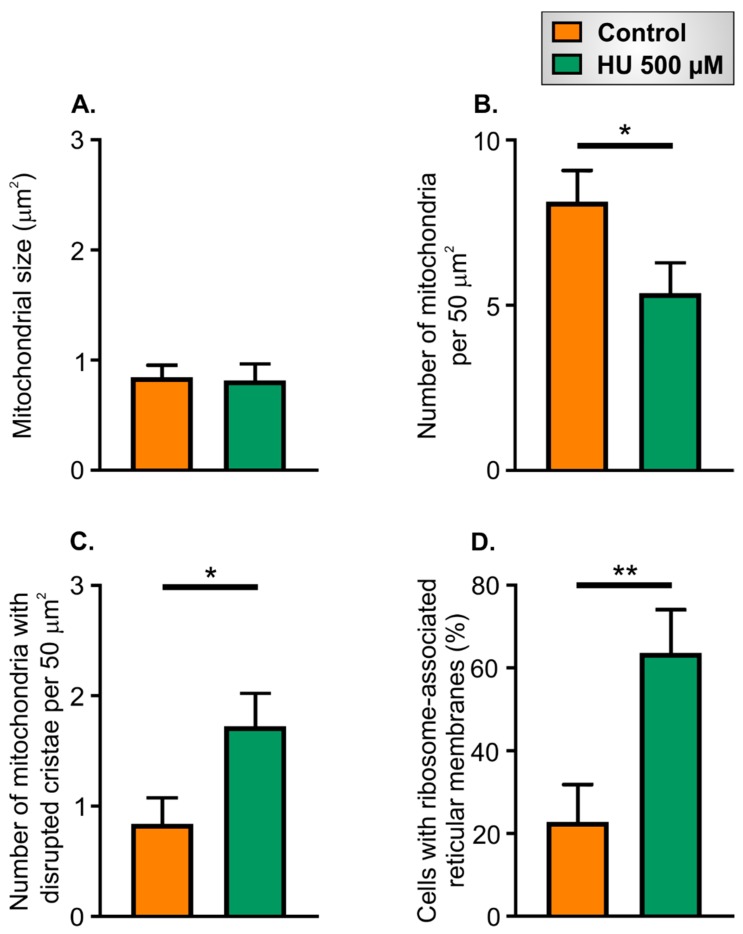
Quantification of ultrastructural aspects of control and HU-treated NRVMs. Panel (**A**) shows the mitochondrial area. Panel (**B**) indicates mitochondrial number per 50 µm^2^. Panel (**C**) presents the number of mitochondria with disrupted cristae per 50 µm^2^. Panel (**D**) shows the percentage of NRVMs containing ribosome-associated membranes in control and HU-treated cells. The data are presented as mean ± S.E.M. and were analysed using unpaired *t*-tests. * and ** denote *p* < 0.05 and *p* < 0.01, respectively. The data are representative of 22 images obtained for each condition.

The mitochondrial size was similar in control and HU-treated NRVMs (Figure 10A), but there was a significant decrease in the density of mitochondria in the cells that had been incubated with HU (Figure 10B). Moreover, there was a significant increase in the number of mitochondria with disrupted cristae in the HU-treated NRVMs (Figure 10C). These data are consistent with the hypothesis that the HU-treated cells had fewer mitochondria overall, and a higher proportion of the mitochondrial population was damaged/dysfunctional [38]. The proportion of NRVMs with ribosome-associated reticular membranes was approximately 3-fold higher in HU-treated cells compared to control (Figure 10D).

**Figure 11:**
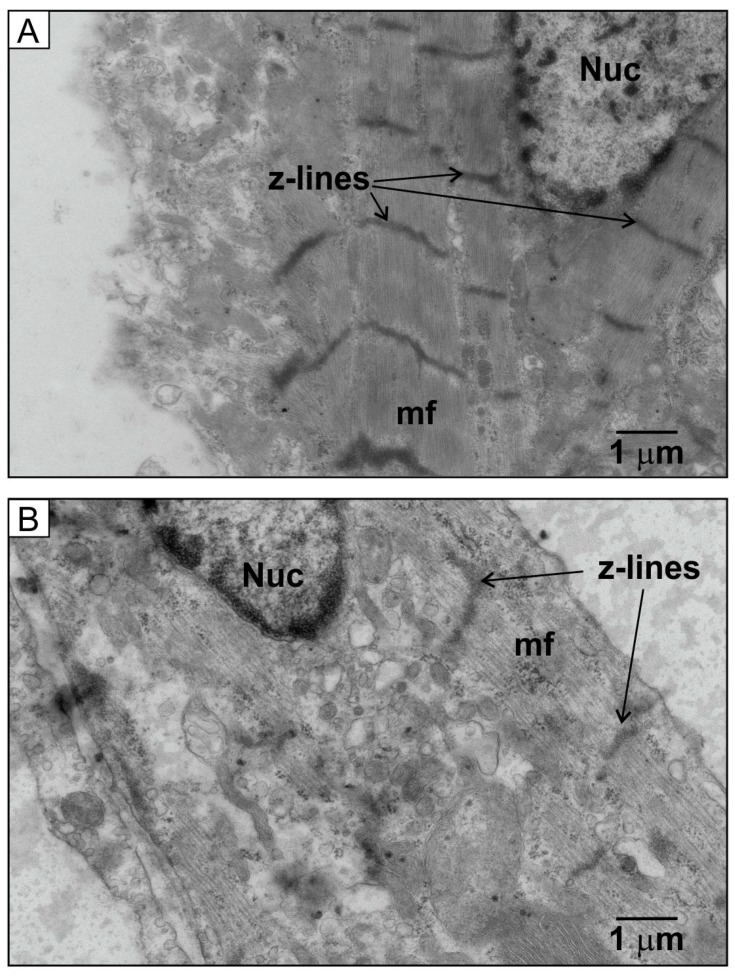

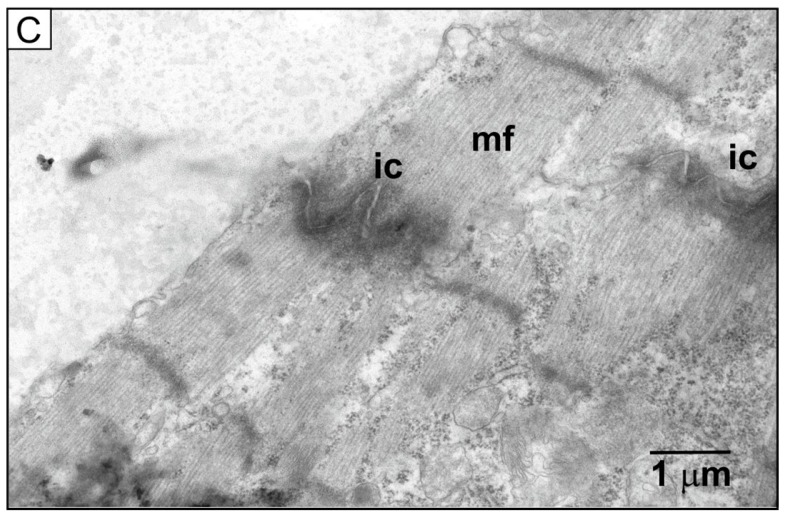
TEM images of control and HU-treated cells. Panel (**A**) shows a TEM image of control NRVMs on day 7, illustrating the presence of myofilaments (mf) and z-lines. Panel (**B**) is a representative TEM image from 500 µM HU-treated cells on day 7, which represents the majority of cell sections obtained (25 out of 30 images) showing fewer myofilaments and less obvious z-lines. Panel (**C**) is an image from an HU-treated cell that displayed some myofibres, evident z-lines, and intercalated discs (ic) (5 out of 30 images). The data are representative of 30 images obtained for each condition.

The HU-treated NRVMs had less evident myofibres and z-lines compared to control cells (Figure 11). NRVMs do not have the same organisation as fully differentiated adult cardiomyocytes [39], so their myofibres can appear to run in non-parallel directions ([39] and Figure 11A). However, the TEM images clearly indicated features within the control NRVMs, such as dense bundles of myofibres and prominent z-lines (Figure 11A). In contrast, the HU-treated NRVMs had fewer organised myofibre bundles and less prominent z-lines (Figure 11B). It is important to point out that the organisation of the HU-treated cells was not entirely disrupted, and some TEM sections with aligned myofiber bundles and corresponding z-lines were observed (Figure 11C), as well as other structures, such as intact nuclear envelope (Figure 11B) and intercalated discs (Figure 11C). The HU treatment, therefore, did not cause a general breakdown of the NRVM structure.

**Figure 12:**
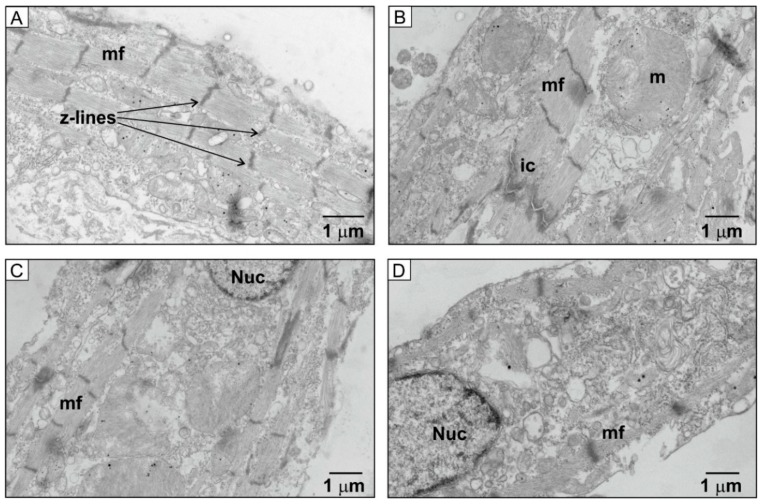
TEM images of control and D-galactose-treated cells. Panels (**A**–**D**) show TEM images of D-galactose-treated cells on day 7, illustrating the presence of myofilaments (mf), z-lines, mitochondria (mi) and intercalated discs (ic). The data are representative of 35 images obtained for each condition.

To investigate whether the effects of HU treatment on NRVM ultrastructure could be observed with another agent that has also been suggested to acutely recapitulate aspects of ageing, cells were incubated with D-galactose for 7 days. As with HU-treated NRVMs, cells that had been incubated with D-galactose appeared to have less dense myofibres and fewer z-lines (Figure 12). Similar to HU-treated NRVMs, myofibres and z-lines could be found in the D-galactose-treated cells (Figure 12A), along with intercalated discs (Figure 12B) and intact nuclei (Figure 12C,D). However, the majority of NRVMs that had been incubated with D-galactose had fewer myofibres that also appeared less well organised compared to control cells (c.f. Figure 9A, Figure 11A and Figure 12).

## 3. Discussion

Investigating ageing using longitudinal studies with naturally-aged animals or progeroid models requires a long lead-time, considerable administrative work, is expensive, and may be prone to co-morbid factors that influence the health of animals. Whilst the use of aged animals and tissues is an optimal approach to study ageing, alternative systems may allow complementary studies at a much faster rate and lower cost. An alternative approach that has been used for some cell types is ageing of cells from young and old animals in vitro [40]. However, adult cardiomyocytes do not survive for long in culture and, therefore, cannot be readily used for longitudinal ageing studies. NRVMs will survive in ex vivo culture for much longer than adult myocytes, but again these cells will not persist indefinitely. The present study sought to investigate whether it is possible to study the phenotype of aged cardiomyocytes without going through a protracted culturing process. If it was possible to recapitulate the morphological and functional changes that occur in aged cells, then investigations could be performed without requiring animals to be housed for long periods, thereby reducing experimental time and waste.

A range of in vitro ageing approaches has been developed over the past decades, including the use of cultured cells, organoids and modelling [40,41,42]. In particular, HU and D-galactose have been used to induce accelerated ageing in mice models, and have been shown to cause systemic effects such as neurological and behavioural changes [43,44] and loss of muscle strength [45], which are consistent with ageing outcomes. To date, HU has not been used to establish an acute ageing model with cardiac myocytes. Numerous studies have investigated cellular responses to HU and D-galactose. A common finding is that both compounds lead to cellular changes via free radicals or reactive oxygen species. Furthermore, they have been reported to cause ageing-related effects, including accumulation of senescence-associated-*β*-galactosidase staining, advanced glycation end products, DNA damage, telomere shortening, activation of signalling pathways, mitotic arrest, and senescence [6,46,47,48].

A key aspect of any ageing model is that it mimics, at least to a large degree, the phenotypic changes observed during natural ageing. This study examined several aspects of cardiomyocyte function to explore the validity of an ageing model arising from the acute treatment of cells with HU. A number of experimental outcomes observed in the HU-treated NRVMs concur with known changes in aged cardiomyocytes, including decreased ability follow electrical pacing (Figure 2B), increased alternans (Figure 2C), decreased cytosolic Ca^2+^ clearance (Figure 4), reduced mitochondrial membrane potential (Figure 6), elevated ROS production (Figure 7), and ultrastructural changes (Figure 9, Figure 10, Figure 11 and Figure 12) [49,50].

Of particular significance are the changes in Ca^2+^ signalling and the inability of the cells to faithfully follow EFS pacing. Control cells responded to EFS with regular responses on days 1, 4 and 7 of culture. Whereas, HU treatment reduced the percentage of cells that showed regular responses and increased the number of cells showing alternans on days 4 and 7 (Figure 2). Alternans are known to be important for the development of arrhythmia [51] and have been associated with cardiac ageing [52,53,54]. Moreover, a number of HU-treated cells were observed not to follow the 2 Hz EFS with neither regular responses nor alternans. Instead, they missed some EFS pulses altogether and responded at a lesser frequency. An example of this is depicted in Figure 1A (right-hand trace obtained from NRVMs treated with 500 µM HU: note that the cell showed fewer EFS-induced Ca^2+^ transients than the control although the pacing frequency was the same). The fact that the occurrence of alternans and the inability to electrically pace the cells were not observed at day 1 (after 24 h of HU treatment) indicated that the effect of HU was not simply to dysregulate normal EC-coupling in the NRVMs.

The ultrastructural changes in the HU-treated NRVMs also point to critical alterations in the ability of cardiomyocytes to function when aged. In particular, there was a reduction of mitochondrial density and an increase in the proportion of mitochondria with disrupted cristae in the HU-treated cells (Figure 9 and Figure 10). Previous studies have reported variable alterations in the number and size of cardiomyocyte mitochondria in conditions such as atrial fibrillation [25,55], and during ageing [56,57]. Disruption of mitochondrial cristae has been previously observed in aged animals. Indeed, mitochondrial cristae were found to be concentrated in some areas within aged mitochondria and absent from other areas [56,58]. Another structural feature that was obvious in the HU-treated NRVMs was membranous cytoplasmic bodies known as lipofuscin granules (Figure 9). These structures are most likely formed by lipid/ganglioside accumulations resulting from defective lipid metabolism. They are a type of lipid storage product that is found in old cells and is a prominent marker for some lysosomal storage diseases [37]. Overall, the phenotypic and functional changes with the HU-treated NRVMs resembled established traits in cardiomyocytes from naturally-aged animals.

It had previously been shown that the mitochondrial membrane potential collapses during ageing, calorific restriction and p53-induced cell senescence [38,59,60,61], which agrees with the results obtained from HU-treated NRVMs (Figure 6). Of note, depolarised mitochondria may produce more ROS [62], which is consistent with the observation that HU-treated NRVMs showed a higher inducible ROS production compared to control cells (Figure 7). Ageing has been linked to increased ROS levels [61,63,64,65]. An age-dependent increase in lipofuscin granules and ROS levels, coupled with a decrease in the mitochondrial membrane potential was observed after keeping NRVMs in culture for up to 3 months [38,66]. A change in mitochondrial membrane potential and the increased ROS levels can affect cellular Ca^2+^ homeostasis [61,63,67,68]. In this study, basal ROS production was similar in control and HU-treated cells (Figure 7). Our interpretation is that ROS production/cellular ROS buffering were close to the physiological range in the HU-treated cells until the cells were given an extra stress (antimycin; Figure 7), and under those conditions, the mitochondria became fully depolarised (Figure 5) so that increased ROS production was evident.

Whilst many of the functional and phenotypic characteristics of HU-treated NRVMs examined in this study resembled changes observed in naturally aged cardiomyocytes, there are some differences. For example, the HU-treated cells showed a significant increase in the proportion of cells with ribosome-associated reticular membranes (Figure 9 and Figure 10). These observations suggest that HU-treated NRVMs have a higher level of protein synthesis compared to the control cells. However, protein synthesis and the number of membrane-associated ribosomes are usually reduced with increasing age in cardiac myocytes [69]. It is plausible that the increase in ribosome-associated reticular membranes represented an HU-induced cellular stress response [70,71]. An increase in autophagosome number was also observed in the HU-treated NRVMs (Figure 8 and Figure 9). Many studies have found that autophagy in cardiac myocytes decreased with age and that increasing the rate of autophagy could alleviate the effects of ageing [72,73,74]. However, there have also been contrasting findings showing that autophagy is upregulated in aged cells [75]. Further studies will be needed to confirm the utility of acute ageing models, in particular alongside conventional ageing experiments. In addition to refining such culture models and investigating cellular changes, it will be necessary to explore the effects of acute ageing protocols on the relationship between arrhythmia risk markers, such as the QT interval [76]. Future studies should also consider systemic effects such as changes in arterial stiffness, which is a marker of both arterio- and atherosclerosis and is related to oxidative stress [77]. Unwarranted interactions of treatments such as HU with tissues may potentially limit their usefulness. In this respect, it is interesting to note that acute myocardial infarction was reported during chemotherapy involving HU in patients without prior heart disease, related to coronary artery spasm, endothelial injury as well as coagulation disorders [78].

In summary, the treatment of NRVMs with HU recapitulates several functional and phenotypic changes associated with the natural ageing of cardiomyocytes [50]. In this study, we specifically observed impaired calcium signalling, altered mitochondrial metabolism, enhanced ROS production, elevated autophagy, abundance of reticular membranes decorated with ribosomes, and disrupted cell structures in HU- and D-galactose treated myocytes. Whilst there may not be an absolute overlap between the features of naturally aged cardiomyocytes and HU-treated cells, there are a number of key parameters that are found in both situations. It is notable that although longitudinal or comparative studies using young versus aged animals are generally considered to be optimal for investigating ageing, they do not always reach the same conclusions, particularly with respect to changes in the heart [2]. Chemical models that evoke ageing-relevant changes, therefore, deserve further attention as they may provide tractable and relatively inexpensive systems for analysis of cellular systems and their dysfunctions that lead to morbidity and mortality.

## 4. Materials and Methods

### 4.1. Preparation of NRVMs

Neonatal rat ventricular myocytes (NRVMs) were prepared as previously described [79], following the United Kingdom Animals Scientific Procedure Act 1986 and approved by the Institutional Animal Care and Use Committee of The Open University. NRVMs were kept in a humidified cell culture incubator at 37 °C, 5% CO_2_ in high glucose DMEM:M199 medium (4:1 mix; Sigma) supplemented with 10% horse and 5% fetal calf serum (Gibco, UK). 1 mM Na^+^ pyruvate (Gibco, UK), 1 mM MEM Non-essential amino acids (Gibco, UK), 1% antibiotic/antimycotic (Gibco) and 3 µM cytosine β–d-arabinofuranoside (Sigma, Welwyn Garden City, UK). NRVMs were plated on 16 mm diameter glass coverslips coated with laminin (Sigma-Aldrich, Poole, UK). The medium was replaced 24 h after plating to remove debris and dead cells. Two days after plating, when the cells started beating, the medium was replaced with a control medium (no additions) or medium containing 50 or 500 µM hydroxyurea (HU, Sigma-Aldrich, Poole, UK) and replaced thereafter every three days.

### 4.2. Ca^2+^ Imaging

The loading buffer for the live-cell experiments was sHBSS, prepared from HBSS (Gibco), supplemented with 25 mM HEPES (Sigma), with the pH was adjusted to 7.4. For Ca^2+^ imaging experiments, NRVMs were loaded with 10 µM of the Ca^2+^-sensitive indicator Cal-520 AM (Stratech, Ely, UK) in the presence of 0.1% Pluronic F127 (Life technologies, Renfrewshire, UK) in sHBSS for 30 min at room temperature. The indicator was washed off and replaced with fresh sHBSS, and the cells were kept for a further 30 min in the dark to de-esterify the indicator. sHBSS was replaced again just prior to the imaging experiments. Where indicated, caffeine (Sigma-Aldrich, Poole, UK) was added on the stage, after recording the basal fluorescence. Imaging experiments were performed using a Leica DMI6000 widefield epifluorescence imaging system with a 20× air objective (NA 0.4) and Leica AFM software. Time-series image sequences were collected at 12–25 frames per second. Regions of interest for analysis were set using ImageJ. Following background subtraction, changes in the fluorescence were analysed using GraphPad Prism 6.0. For electrical field stimulation (EFS), sine wave pulses of a current of ~4.2 −8 mAmps, 60 V and 10 ms duration were applied using a Grass Instruments SD9 simulator with platinum electrodes placed at side of the imaging chamber. The stimulation frequency was set at 2 Hz.

### 4.3. Measurement of the Mitochondrial Membrane Potential

NRVMS were loaded with 50 µM JC-10 (Stratech, Ely, UK) and 100 nM MitoTracker Deep Red (Life Technologies, Renfrewshire, UK) for 30 min at room temperature in sHBSS (specified above). JC-10 was excited using 450–490 nm (green) and 590–650 nm (red), and the emission recorded above 650 nm. MitoTracker was excited at 579 nm, and the emission measured above 650 nm. Where indicated, antimycin (5 µM) was added to cells on the microscope stage to depolarise mitochondria. Image collection and analysis were performed as described above.

### 4.4. Measurement of ROS Levels

NRVMS were loaded with 50 µM ROS Brite (Stratech, Ely, UK) for 30 min at room temperature in sHBSS (specified above). ROS Brite was excited at 488 nm, and emission was measured above 515 nm. Where indicated, ROS production was induced by the addition of 10 µM antimycin 10 s after starting the imaging experiment. Image collection and analysis were performed as described above.

### 4.5. Autophagy Assay

Autophagy was measured using a CYTO-ID autophagy kit (Enzo Life Sciences, Exeter, UK). The CytoID detection reagent was diluted in the assay buffer, and NRVMs were loaded for 30 min at 37 °C before being washed and imaged on a fluorescence microscope. The CytoID reagent was excited at 488 nm, and emission was measured above 515 nm. Image collection and analysis were performed as described above.

### 4.6. Transmission Electron Microscopy

NRVMs were prepared for electron microscopy according to standard TEM protocols [80] and cut into ~70 nm ultrathin sections. These were imaged on a JEOL 1400 TEM (JEOL, Japan) using an 80 kV electron beam and a spot size of 2 µm. Images were taken with an XR60 camera (Advance Microscopy Techniques, Woburn, MA, USA) using AMTV600 software (Advance Microscopy Techniques, Woburn, MA, USA). Image analysis was performed using Image J.

### 4.7. Experimental Repeats and Statistical Analysis

Pooled NRVM cultures were obtained by combining the cardiac cells from all the neonates available in a rat litter. The number of neonates within a litter varied from 4–14. Cells from the NRVM pool were plated on coverslips prior to use, as described above. Since all the cells on coverslips experienced the different conditions used in this study (e.g., control or HU-treated), a coverslip was considered as the experimental unit for this study. For most live-cell studies, data were obtained from multiple adjacent cells or by scanning cells in different regions of a coverslip. The data arising from a coverslip (whether obtained from a single cell or averaged from multiple cells) were used as a single data point for statistical analysis. For EM analysis, quantifications were made on the individual images, and the data arising from a single image were used as a single data point for statistical analysis. The data presented in the figures represent the average ± S.E.M. of the response observed from replicate coverslips. The number of replicate coverslips used in each experiment is stated in the figure legends. The results shown in Figure 1, Figure 2, Figure 3, Figure 4, Figure 6, Figure 7 and Figure 8 were obtained by combining the data obtained from three different NRVM preparations. Figure 1, for example, involved a total of nine coverslips, with three coverslips being used from each of the three NRVM preparations. The results shown in Figure 5, which was a proof of principle experiment, were obtained from one NRVM preparation. The EM images (Figure 9, Figure 10, Figure 11 and Figure 12) were obtained from two NRVM preparations. The data were analysed either with an unpaired *t*-test or a One-way ANOVA, as specified in the figure legends, using GraphPad Prism software.
